# Simultaneous occurrence of fibrillary glomerulonephritis and renal lesions in nonmalignant monoclonal IgM gammopathy

**DOI:** 10.1186/s12882-015-0198-y

**Published:** 2016-02-18

**Authors:** Chung-Kuan Wu, Jyh-Gang Leu, An-Hang Yang, Der-Cherng Tarng, Hsiang-Yuen Tung, Shou-Shan Chiang

**Affiliations:** Division of Nephrology, Department of Internal Medicine, Shin-Kong Wu Ho-Su Memorial Hospital, 95,Wen Chang Rd., Shih Lin District Taipei, 11101 Taiwan; Institute of Clinical Medicine, National Yang-Ming University, Taipei, Taiwan; School of Medicine, Fu-Jen Catholic University, New Taipei City, Taiwan; Department of Pathology and Laboratory Medicine, Taipei Veterans General Hospital, Taipei, Taiwan; Division of Nephrology, Department of Medicine, Taipei Veterans General Hospital, Taipei, Taiwan

**Keywords:** Fibrillary glomerulonephritis (FGN), IgM monoclonal gammopathy, Membranoproliferative glomerulonephritis

## Abstract

**Background:**

Fibrillary glomerulonephritis (FGN) is a rare primary glomerular disease that seldom coexists with other diseases. Membranoproliferative glomerulonephritis is a pathologic finding of renal lesions associated with IgM-secreting monoclonal proliferations. We present a case study of a patient with unusual simultaneous FGN and IgM-related renal disorder in nonmalignant monoclonal IgM gammopathy.

**Case presentation:**

A 63-year-old male presented with nephrotic syndrome and elevated serum creatinine levels. Laboratory examination revealed elevated levels of serum IgM and low C3 levels. Serum and urine immunofixation electrophoresis showed a monoclonal IgM with a kappa light chain. A bone marrow biopsy revealed less than 5 % bone marrow infiltration by lymphoplasmacytic lymphoma, and a renal biopsy revealed mesangiocapillary glomerulonephritis on light microscopy. Immunofluorescent and immunohistochemical staining indicated granular deposits of immunoglobulin G in the mesangium and granular deposits of immunoglobulin M and κ light chains along the capillary wall. Electron microscopy revealed randomly arranged nonbranching fibrils of approximately 15 nm in diameter in the glomerular mesangium and subendothelial electron-dense deposits. According to these results, we confirmed FGN and membranoproliferative glomerulonephritis, which were attributed to monoclonal IgM deposits.

**Conclusion:**

To the best of our knowledge, this is the first report of simultaneous FGN and membranoproliferative glomerulonephritis in nonmalignant IgM monoclonal gammopathy.

## Background

The term “fibrillary glomerulonephritis” (FGN) was introduced by Alpers et al in 1987 to characterize the glomerular accumulation of nonbranching, randomly arranged fibril, which differ from amyloid deposits in their large size and lack of reactivity to Congo red [1]. FGN is a rare disorder, diagnosed in less than 1 % of renal biopsies and usually presents with renal insufficiency, nephrotic range proteinuria, and hematuria [2].

IgM monoclonal gammopathies can be categorized into symptomatic, asymptomatic Waldenström’s disease, IgM–related disorders, and IgM monoclonal gammopathy of unknown significance (MGUS) [3]. Renal involvement in IgM monoclonal gammopathy is typically found in patients with the malignant disease, Waldenström’s macroglobulinemia, which is associated with B-cell lymphoproliferative disorder [4]. Renal lesions include the deposition of monoclonal IgM and light chains on the mesangium and glomerular capillary wall [5, 6]. In patients with nonmaligant IgM monoclonal gammopathy, renal involvement has seldom been reported [7].

We present a case report of a patient with nonmalignant IgM/κ gammopathy who developed nephrotic syndrome associated with FGN and the renal deposition of IgM and κ light chains.

## Case presentation

A 63-year-old man presented at our nephrologic outpatient clinic with progressive bilateral leg edema and foamy urine, which he had experienced for 1 month. He was hospitalized for alcoholic pancreatitis in 1999 but not regularly followed up by our hospital after discharge.

Physical examination of the patient revealed a blood pressure of 150/85 mmHg, blood temperature of 36.5 °C, and pulse rate of 78 beats/minute; the grading scale for pitting edema was 3+. The laboratory results were as follows: blood urea nitrogen, 26 mg/dL; serum creatinine, 1.8 mg/dL; albumin, 3.1 g/dL; hemoglobin, 12.4 g/dL and platelets, 212 × 10^3^ / uL. Urinalysis revealed 2+ occult blood, 3+ protein, and 5-7 red blood cells/high power field; the 24-h protein excretion was 5.7 g/day. Serum immunoglobulin (Ig) and serum complement tests yielded high IgM (498 mg/dL), low C3 and IgG (73 and 688 mg/dL, respectively), and normal IgA and C4 levels. Serum and urine immunofixation electrophoresis showed a monoclonal IgM-bearing kappa light chain. The urinary Bence Jones protein was negative. The rheumatoid factor, antinuclear antibody, cryoglobulin, and other autoantibodies were negative. Serum antibodies against HIV, hepatitis B and C were all negative. A bone marrow biopsy revealed hypocellularity with normal maturation of myeloid series, and less than 5 % of the cells had positive immunohistochemical staining of CD138/syndecan-1 plasma cells.

Renal sonography showed that both kidneys were enlarged. Chest and abdominal computerized tomography ruled out organomegaly and lymphadenopathy. A whole body bone X-ray revealed no lytic bone lesions.

**Fig. 1:**
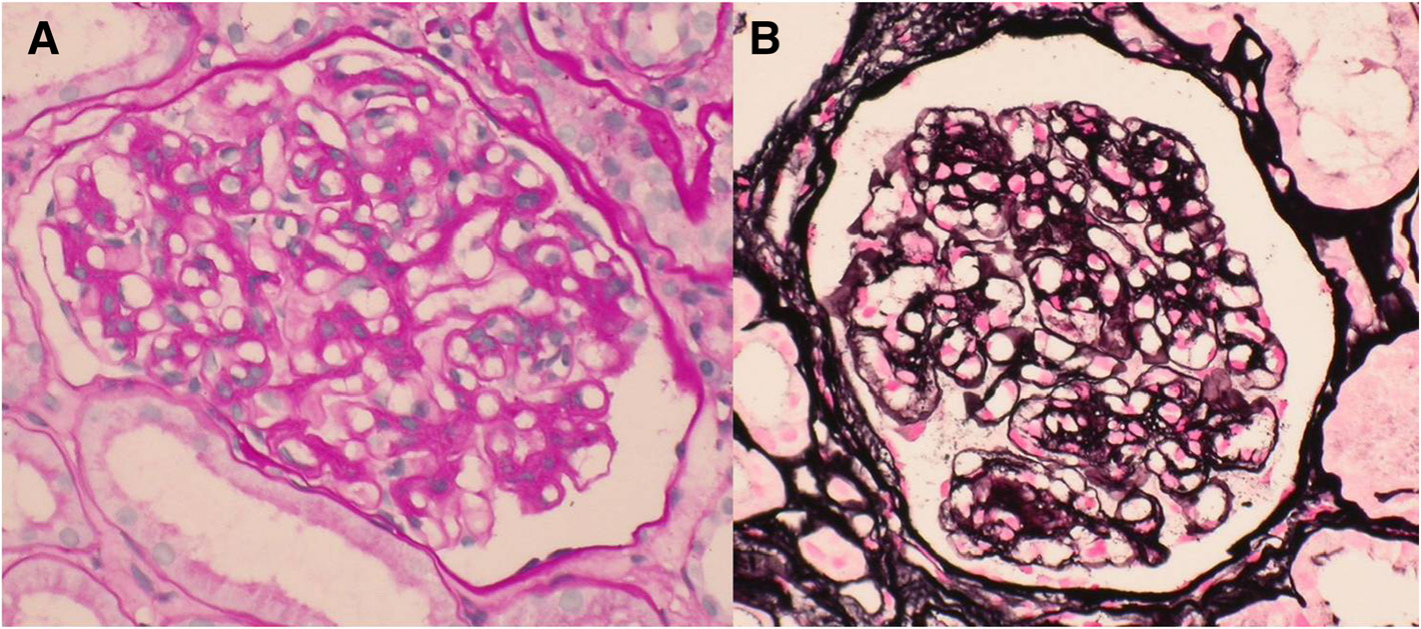
Light microscopic features of membranoproliferative glomerulonephritis. (**a**) The mesangium is expanded and the glomerular capillary walls appear thickened (periodic acid-Schiff). (**b**) Glomerular capillary walls exhibit thickened and segmental double contours (methenamine silver)

Light microscopy of the renal biopsy revealed nodular segmental glomerulosclerosis with mesangial cell proliferation and mesangial matrix expansion (Fig. 1a) in 9 of the 11 glomeruli; the other 2 glomeruli are global scleroses. In addition, focal segmental double-contoured capillary walls were observed, and mild tubular atrophy, interstitial fibrosis, and mononuclear cell infiltration were found (Fig. 1b). Congo red staining was negative.

**Fig. 2:**
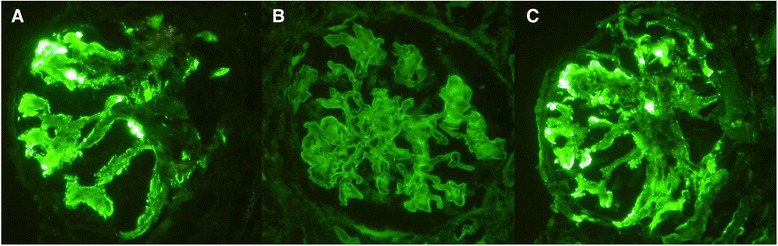
Immunofluorescent staining on fibrillary glomerulonephritis and renal involvement of IgM gammopathy. (**a**) Diffuse granular deposits of IgM along the glomerular capillary walls. (**b**) Smudged diffuse granular mesangial deposits of IgG. (**c**) Diffuse segmental granular deposits of C3 on the mesangium and along the glomerular capillary walls

**Fig. 3:**
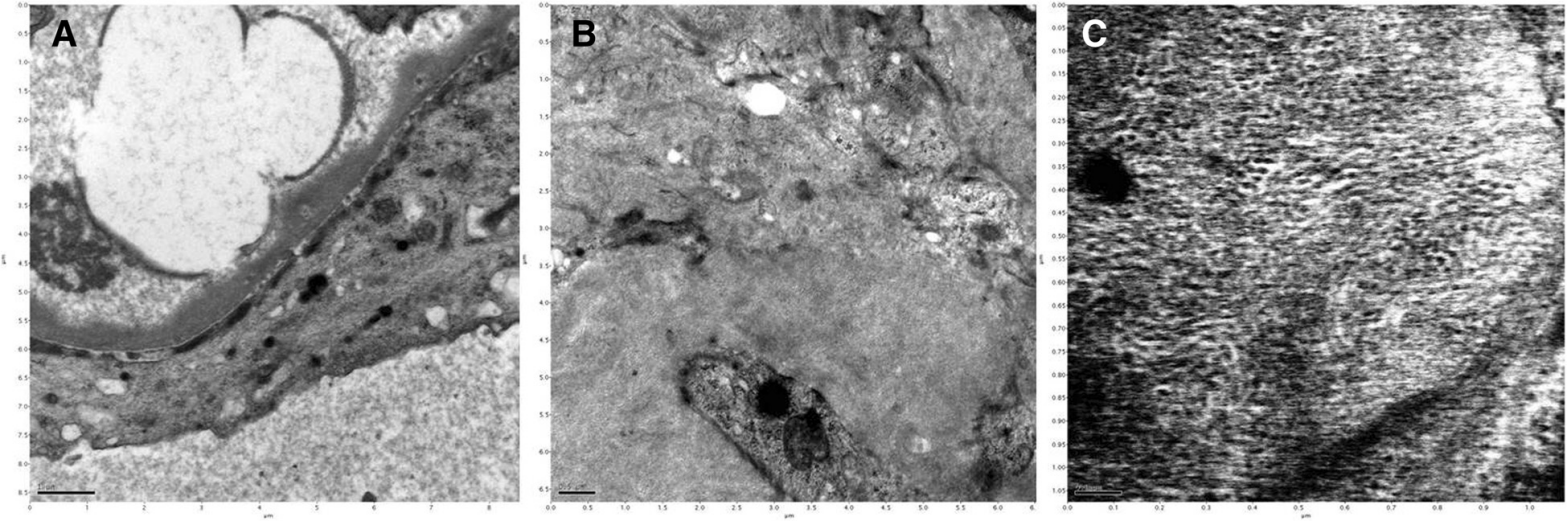
Electron microscopic features of subendothelial deposits and fibrillary glomerulonephritis. (**a**) Electron micrograph image revealing granular subendothelial deposits and small intracapillary deposits. (**b**) Electron micrograph image showing infiltration of fibrillar materials in the mesangium. (**c**) Irregular accumulations of randomly arranged nonbranching fibrils in the mesangium

We performed immunofluorescence studies, observing a strong positive staining for IgM with a diffuse, global, granular, and capillary pattern (Fig. 2a) ; positive staining for IgG with a diffuse, granular, and mesangial pattern (Fig. 2b); and positive staining for C3 with a diffuse, segmental, and granular pattern in the mesangium and along the capillary wall (Fig. 2c). Immunohistochemistry of the light chains revealed κ positive along the capillary wall. Electron microscopy showed podocyte foot process effacement and microvillus transformation. Electron-dense materials were deposited in the subendothelial area and limited intracapillary monoclonal deposits were observed (Fig. 3a). Irregular accumulations of randomly arranged nonbranching fibrils, approximately 15 nm in diameter, were found in the glomerular mesangium (Fig. 3b, c). These findings confirmed the diagnosis of FGN with membranoproliferative glomerulonephritis in nonmalignant IgM monoclonal gammopathy.

The patient was treated initially with diuretics, angiotensin receptor blockers, 0.5 mg/kg of prednisolone per day, and 1.5 mg/kg of cyclophosphamide per day. Because steroid-induced diabetes occurred, we halved the steroid dose within the first 3 months. After 6 months, serum creatinine levels were maintained at 1.8 mg/dL and the 24-h protein excretion decreased to 1.8 g/day. 1 year later, the patient experienced herpes zoster; therefore, we discontinued cyclophosphamide treatment. However, the patient progressed to stage 4 chronic kidney disease the second year after diagnosis and advanced to end-stage renal disease (ESRD) without dialysis the third year after diagnosis.

## Conclusions

FGN, first described by Rosenmann and Eliakim in 1977 [8], is a rare primary glomerulonephritis diagnosed by the ultrastructural features of nonbranching random fibrils measuring 10-30 nm in diameter. The fibrils are deposited in the mesangium, the glomerular basement membranes, or both. The light microscopic findings are highly variable, and most exhibit mesangial expansion and hypercellularity with or without the duplication of glomerular basement membranes. Immunofluorescence staining is almost consistently positive for IgG, C3, and both κ and λ chains, indicating polyclonal deposits. Weak staining for IgM, IgA, and C1q has been reported in a few cases [2, 9, 10].

Our patient with FGN exhibited various atypical characteristics, including a strong positive immunofluorescent intensity of IgM along the glomerular capillary wall; the presence of IgM κ, as detected by immunofixation electrophoresis, and hypocomplementemia. In a large single-center series of 66 FGN cases, 47 % of the cases were weakly positive for IgM according to the immunofluorescence stain, and the mean intensity of the positive cases was 1.0+. Of the 11 patients exhibiting a positive M-spike, all were IgG with or without light chains according to immunofixation electrophoresis. Only one patient in this study had hypocomplementemia [11]. Only one case of FGN with unusual IgM deposits and hypocomplementemia has been reported in the English literature. A 12-year-old girl who presented with nephrotic-nephritic syndrome had IgM deposits in the mesangium and randomly arranged fibrillary electron-dense deposits in the mesangium, and the subendothelial and subepithelial area of capillary loops. However, the patient did not have elevated levels of serum IgM or positive IgM detected by urine protein electrophoresis [12].

At an international workshop on Waldenström’s macroglobulinemia, 4 categories of IgM monoclonal gammopathies were proposed. Patients with bone marrow infiltration by lymphoplasmacytic lymphoma who (1) might or might not (2) have symptoms caused by IgM are categorized as having symptomatic or asymptomatic Waldenström’s disease, respectively. (3) Patients with symptoms attributable to IgM but with no bone marrow infiltration are classified as having IgM-related disorder. (4) Patients with IgM monoclonal gammopathy but no bone marrow infiltration or IgM-related symptoms are classified as having MGUS [3].

The incidence of renal complications of malignant IgM-secreting proliferation is rare, and renal involvement in IgM monoclonal disorders is generally described in patients with Waldenström’s macroglobulinemia [6]. The typical pathologic findings of Waldenström’s macroglobulinemia-related nephropathies include intracapillary deposits of IgM with or without cryoglobulinemia, light chain amyloidosis, and infiltration of the interstitium by neoplastic lymphoplasmacytic cells [5]. Glomerular involvement was noted in some patients with typical mesangioproliferative or mesangiocapillary glomerulonephritis with IgM deposits in the mesangium and/or along the glomerular basement membrane [13]. Upon immunofluorescence stain, the deposits of IgM and the single light chain isotype were consistent with circulating M components. Electron microscopy revealed that the subendothelial deposits contain nonamyloid fibrillar materials [14].

Our patient had renal lesions associated with IgM-secreting monoclonal proliferations but did not fulfill the criteria for having Waldenström’s macroglobulinemia. Audard et al. revisited the spectrum of renal lesions occurring in patients with a circulating monoclonal IgM and kidney disease related to B cell proliferation. Of the 14 patients examined, 5 patients had intracapillary IgM monoclonal deposits disease, 3 patients had a neoplastic lymphoplasmacytic infiltration of the interstitium, 3 patients had membranoproliferative glomerulonephritis, 2 patients had amyloidosis, and 1 patient had acute tubular necrosis. According to immunologic data, only 3 patients had low blood complement C3, C4, and CH50 levels; all these patients were diagnosed with Waldenström’s macroglobulinemia [15]. Our patient had circulating monoclonal IgM and a low blood complement C3 level but no bone marrow infiltrations. A kidney disease related to B- cell proliferation is membranoproliferative glomerulonephritis, but unusual nonbranching randomly fibrils is found in the mesangium.

Regarding treatment, although no effective therapy has been established for FGN, some clinicians have treated the disease according to pathologic findings. Cyclosporine has been used successfully to treat patients with a membranous pattern, and rituximab has been used successfully to treat patients with an MPGN pattern [9, 16]. In previous investigations, the prognosis of patients with FGN has been poor, and 40 %-50 % of such patients have developed ESRD within 6 years of presentation [2, 17]. Treatment recommendations for Waldenström’s macroglobulinemia were established in the aforementioned international workshop. Patients with Waldenström’s macroglobulinemia should be treated when their hemoglobin level is less than 100 g/L, their platelet count is less than 100 × 10^9^/L, and they present with bulky disease, symptomatic hyperviscosity, severe neuropathy, amyloidosis, cryoglobulinemia, cold-agglutinin disease, or evidence of disease transformation [3, 6]. Several drugs are used for frontline therapy, such as alkylators (ie, chlorambucil), nucleoside analogues (ie, fludarabine) and monoclonal antibodies (ie, rituximab), and combination therapies include cyclophosphamide, doxorubicin, vincristine, and prednisolone. Other alternative treatments included autologous transplantation, thalidomide or thalidomide plus steroids, and alemtuzumab [6]. Rituximab was suggested for our patient, but his condition did not satisfy the criteria for health insurance payment. Finally, in addition to conservative treatment with angiotensin receptor blocker and diuretic drugs, he was treated with cyclophosphamide and prednisolone. Nevertheless, he progressed to ESRD after 2 years of treatment.

In summary, we report a rare simultaneous occurrence of FGN and membranoproliferative glomerulonephritis attributed to IgM/κ deposits in a patient with nonmalignant IgM monoclonal gammopathy. This patient exhibited a wide spectrum of renal lesions caused by IgM-secreting monoclonal proliferations, and renal prognosis was as poor as that for FGN.

## Consent

Written informed consent was obtained from the patient for publication of this case report and any accompanying images. A copy of the written consent is available for review by the editor of this journal.

## Abbreviations

FGN: fibrillary glomerulonephritis
MGUS: IgM monoclonal gammopathy of unknown significance
ESRD: end-stage renal disease

## References

[1] Alpers CE, Rennke HG, Hopper J, Biava CG (1987). Fibrillary glomerulonephritis: an entity with unusual immunofluorescence features. Kidney Int.

[2] Iskandar SS, Falk RJ, Jennette JC (1992). Clinical and pathologic features of fibrillary glomerulonephritis. Kidney Int.

[3] Owen RG, Treon SP, Al-Katib A, Fonseca R, Greipp PR, McMaster ML, Morra E, Pangalis GA, San Miguel JF, Branagan AR (2003). Clinicopathological definition of Waldenstrom's macroglobulinemia: consensus panel recommendations from the Second International Workshop on Waldenstrom's Macroglobulinemia. Seminars in oncology.

[4] Fonseca R, Hayman S (2007). Waldenstrom macroglobulinaemia. Br J Haematol.

[5] Morel-Maroger L, Basch A, Danon F, Verroust P, Richet G (1970). Pathology of the kidney in Waldenstrom's macroglobulinemia. Study of sixteen cases. N Engl J Med.

[6] Treon SP, Gertz MA, Dimopoulos M, Anagnostopoulos A, Blade J, Branagan AR, Garcia-Sanz R, Johnson S, Kimby E, Leblond V (2006). Update on treatment recommendations from the Third International Workshop on Waldenstrom's macroglobulinemia. Blood.

[7] Ramos R, Poveda R, Sarra J, Domingo A, Carreras L, Grinyo JM (2007). Renal involvement in non-malignant IgM gammopathy. Nephrol Dial Transplant.

[8] Rosenmann E, Eliakim M (1977). Nephrotic syndrome associated with amyloid-like glomerular deposits. Nephron.

[9] Rosenstock JL, Markowitz GS, Valeri AM, Sacchi G, Appel GB, D'Agati VD (2003). Fibrillary and immunotactoid glomerulonephritis: Distinct entities with different clinical and pathologic features. Kidney international.

[10] Bridoux F, Hugue V, Coldefy O, Goujon JM, Bauwens M, Sechet A, Preud'Homme JL, Touchard G (2002). Fibrillary glomerulonephritis and immunotactoid (microtubular) glomerulopathy are associated with distinct immunologic features. Kidney international.

[11] Nasr SH, Valeri AM, Cornell LD, Fidler ME, Sethi S, Leung N, Fervenza FC (2011). Fibrillary glomerulonephritis: a report of 66 cases from a single institution. Clin J Am Soc Nephrol.

[12] Shim YH, Lee SJ, Sung SH (2008). A case of fibrillary glomerulonephritis with unusual IgM deposits and hypocomplementemia. Pediatric nephrology.

[13] Kyle RA, Garton JP (1987). The spectrum of IgM monoclonal gammopathy in 430 cases. Mayo Clin Proc.

[14] Gallo GR, Feiner HD, Buxbaum JN (1982). The kidney in lymphoplasmacytic disorders. Pathology annual.

[15] Audard V, Georges B, Vanhille P, Toly C, Deroure B, Fakhouri F, Cuvelier R, Belenfant X, Surin B, Aucouturier P (2008). Renal lesions associated with IgM-secreting monoclonal proliferations: revisiting the disease spectrum. Clin J Am Soc Nephrol.

[16] Collins M, Navaneethan SD, Chung M, Sloand J, Goldman B, Appel G, Rovin BH (2008). Rituximab treatment of fibrillary glomerulonephritis. Am J Kidney Dis.

[17] Fogo A, Qureshi N, Horn RG (1993). Morphologic and clinical features of fibrillary glomerulonephritis versus immunotactoid glomerulopathy. Am J Kidney Dis.

